# Designed Artificial Protein Heterodimers With Coupled Functions Constructed Using Bio-Orthogonal Chemistry

**DOI:** 10.3389/fchem.2021.733550

**Published:** 2021-08-04

**Authors:** Rachel L. Johnson, Hayley G. Blaber, Tomas Evans, Harley L. Worthy, Jacob R. Pope, D. Dafydd Jones

**Affiliations:** ^1^ Molecular Biosciences Division, School of Biosciences, Cardiff University, Cardiff, United Kingdom; ^2^ The Henry Wellcome Building for Biocatalysis, Exeter University, Exeter, United Kingdom

**Keywords:** genetic code expansion, bioorthogonal chemistry, azide alkyne cycloaddition, artificial protein oligomer, protein design and engineering, fluorescent proteins, energy transfer

## Abstract

The formation of protein complexes is central to biology, with oligomeric proteins more prevalent than monomers. The coupling of functionally and even structurally distinct protein units can lead to new functional properties not accessible by monomeric proteins alone. While such complexes are driven by evolutionally needs in biology, the ability to link normally functionally and structurally disparate proteins can lead to new emergent properties for use in synthetic biology and the nanosciences. Here we demonstrate how two disparate proteins, the haem binding helical bundle protein cytochrome *b*_562_ and the β-barrel green fluorescent protein can be combined to form a heterodimer linked together by an unnatural triazole linkage. The complex was designed using computational docking approaches to predict compatible interfaces between the two proteins. Models of the complexes where then used to engineer residue coupling sites in each protein to link them together. Genetic code expansion was used to incorporate azide chemistry in cytochrome *b*_562_ and alkyne chemistry in GFP so that a permanent triazole covalent linkage can be made between the two proteins. Two linkage sites with respect to GFP were sampled. Spectral analysis of the new heterodimer revealed that haem binding and fluorescent protein chromophore properties were retained. Functional coupling was confirmed through changes in GFP absorbance and fluorescence, with linkage site determining the extent of communication between the two proteins. We have thus shown here that is possible to design and build heterodimeric proteins that couple structurally and functionally disparate proteins to form a new complex with new functional properties.

## Introduction

Protein oligomerisation, commonly referred to as protein quaternary structure, is the association of specific individual polypeptide chains through defined intermolecular interactions to form a single multimeric complex (Goodsell and Olson, 2000; Nooren and Thornton, 2003; Ali and Imperiali, 2005). So prevalent is oligomerisation in nature, protein oligomers are more common than their monomeric counterparts, at least in the protein data bank (Goodsell and Olson, 2000; Ali and Imperiali, 2005). Protein oligomers can comprise purely of non-covalent intermolecular interactions or also utilise inter-subunit covalent crosslinking, predominantly via disulphide bridges. Oligomerisation is largely seen as beneficial by reducing surface residues’ (especially hydrophobics) exposure to solvent, resulting in a lower surface area to volume ratio leading to improved stability against degradation and aggregation (Larsen et al., 1998; Ali and Imperiali, 2005; Gwyther et al., 2019). Crucially, oligomerisation also leads to functional features not available in monomers; they locally concentrate multiple active sites resulting in improved activity and enabling functional cooperativity whereby synergy [communication] between each polypeptide unit can positively or negatively regulate activity or even lead to new properties (Goodsell and Olson, 2000; Gwyther et al., 2019).

Given the benefits of protein oligomerisation, protein designers and engineers have sort to address this area by constructing bespoke, artificial protein oligomeric systems (Oohora and Hayashi, 2014; Norn and Andre, 2016; Kobayashi and Arai, 2017; Ljubetic et al., 2017; Gwyther et al., 2019) ranging from simple dimers (Mou et al., 2015; Fallas et al., 2017) to higher order supramolecular structure (Thomson et al., 2014; Gonen et al., 2015; Butterfield et al., 2017). The main problem with these systems is that they lack functional synergy or even function beyond directing the assembly process. Thus, such systems tend to manifest the basic functional properties of their starting components. Recently, we have demonstrated functional synergy between designed artificial dimers (Worthy et al., 2019; Pope et al., 2020). Using computational approaches, we identified mutually compatible interfaces between various β-barrel fluorescent proteins and stabilised the interaction by genetically encoded click chemistry. Our approach allowed us to generate both symmetrical and non-symmetrical dimers together with homo and heterodimers that displayed either positive or negative functional synergy. While dimers represent the simplest protein oligomeric unit, they are the most frequently observed structural form in nature, with homo-dimers (comprising of the same polypeptide) dominating over hetero-dimer (composed of two different polypeptides) (Goodsell and Olson, 2000; Marianayagam et al., 2004; Mei et al., 2005).

The next challenge arguably involves the generation of heterodimers from structurally and functionally diverse proteins. Heterodimers are potentially rich in new functional features as they have the potential to combine drastically different and disparate functions leading to new emergent properties. Here we aim to take the next step in dimer construction and demonstrate that it is feasible to design and build intimately linked heterodimers comprised of structurally and functionally disparate proteins by linking a helical bundle protein to a β-barrel protein using a combination of computational protein design and bio-orthogonal chemistry. While bioorthogonal chemistry has been used previously to link different proteins together (Hatzakis et al., 2006; Eger et al., 2010; Hudak et al., 2012; Schoffelen et al., 2013; Torres-Kolbus et al., 2014; Kim et al., 2016; White and Bode, 2018), little effort is given to predicting compatible interfaces and residues pairs so the individual proteins generally remain functionally and structurally distinct. Furthermore, extended chemical linkers are routinely used as part of the chemical coupling process resulting in spatially separated protein units and preventing any interactions forming that normally comprise natural protein dimers (Hudak et al., 2012; Kim et al., 2012; Schoffelen et al., 2013; White and Bode, 2018). Thus, there is limited benefit above that of traditional genetic fusion approaches. Some rely on natural amino acid chemistry inherent to one partner protein to facilitate linkage (Hatzakis et al., 2006; Schoffelen et al., 2013; Torres-Kolbus et al., 2014), which can restrict coupling sites between individual units. To address these issues, we recently developed a computational design approach to facilitate the design of dimers linked by two genetically encoded compatible bio-orthogonal reaction handles (azide and alkyne) (Worthy et al., 2019; Pope et al., 2020). We focused on structurally similar proteins [fluorescent proteins with a common β-barrel architecture] whereby mutually compatible symmetrical dimer interfaces can facilitate construction. Such symmetry will not be available when structurally distinct proteins are used as proposed here and thus poses a greater challenge to predicting mutually compatible coupling sites and thus heterodimer construction.

**FIGURE 1:**
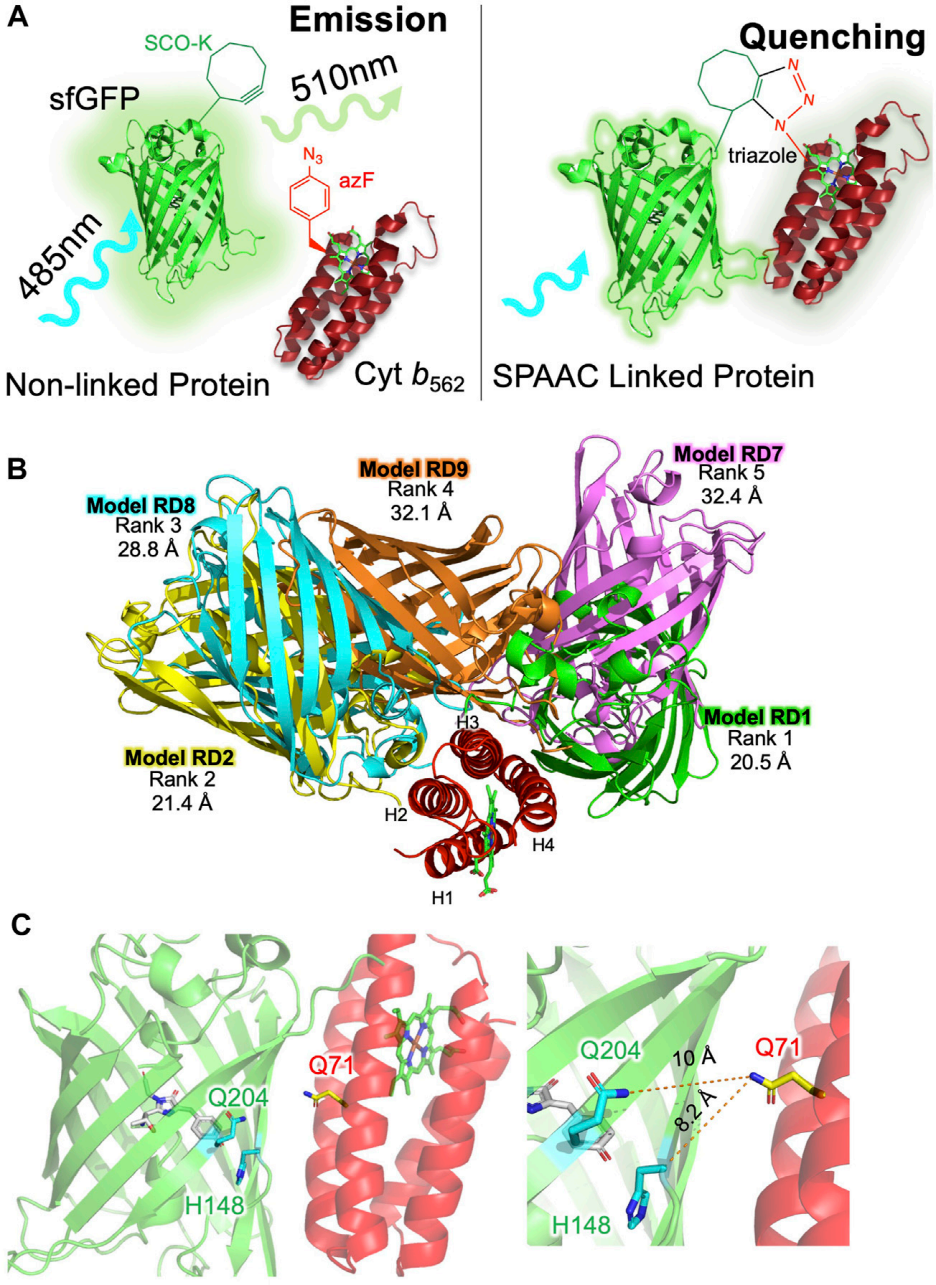
Designing sfGFP-cyt *b*_562_ SPAAC linked heterodimers. **(A)** Basic strategy for constructing heterodimers using SPAAC. As a monomeric protein, sfGFP (green) will excite at ∼485 nm and then emit light at ∼510 nm. On forming a heterodimer, sfGFP fluorescence will predominantly be quenched by cyt *b*_562_ (red). The complex will only persist if SPAAC is successful and a triazole link is formed. **(B)** The top five alignments of sfGFP (various colours) and cyt *b*_562_ (red) ranked according to total energy of interaction by RosettaDock. Distances measured between each model chromophore pair are shown below the rank. Model RD1 has the closest chromophore proximity and lowest total energy. **(C)** Residue selected for mutation to SCO-K (Q204 and H148; green) in sfGFP or AzF (Q71; red) in cyt *b*_562_.

To test our approach, we will use cytochrome *b*_562_ (cyt *b*_562_) as the helical bundle protein and the superfolder version of green fluorescent protein (sfGFP) as the β-barrel protein (Figure 1A). Cyt *b*_562_ is a small 4-helical bundle protein that binds haem tightly but non-covalently (Arnesano et al., 1999) while sfGFP is a directly evolved descendent of the original *Aequorea victoria* GFP (Pedelacq et al., 2006). We have previously linked the function of the closely related homolog of sfGFP, enhanced GFP (EGFP), with cyt *b*_562_ through a directed evolution domain insertion approach (Edwards et al., 2008; Arpino et al., 2012). The domain insertion approach differs significantly from heterodimerization as a single polypeptide unit contains both original proteins; the GFP primary structure is disturbed by insertion of the cyt *b*_562_ sequence within it at the genetic level. One variant demonstrated high energy transfer efficiency (close to 100%). Structural analysis revealed that the two original proteins are close in space within the single polypeptide unit but do not form many distinct inter-unit interactions as would be expected of a true oligomeric system. There was also little change in the inherent function of each domain compared to the starting parent protein suggesting limited synergy between the two.

Here, we used computational approaches to predict compatible interfaces between sfGFP and cyt *b*_562_. We then used 1-to-1 strain promoted alkyne-azide cycloaddition to covalently link the two proteins through a triazole link by genetically encoded incorporation of the azide group (via non-canonical amino acid azidophenylalanine) into cyt *b*_562_ and a strained cyclooctyne group (via the non-canonical amino acid strained-cyclooctyne lysine) in sfGFP. The successfully constructed heterodimers lead to enhanced sfGFP molar absorbance coefficients and were capable of energy transfer from sfGFP to cyt *b*_562_, with linkage site determining transfer efficiency.

## Methods and Materials

### In Silico Docking

The combined ClusPro (Kozakov et al., 2017) and RosettaDock (Leaver-Fay et al., 2011; Alford et al., 2017) approach has been described previously (Worthy et al., 2019). Briefly, the ClusPro Protein-Protein server (https://cluspro.bu.edu) “Dock” function was utilised to predict potential interface sites between proteins. ClusPro simulation was run with cyt *b*_562_ (PDB 1qpu (Arnesano et al., 1999)) as the receptor and sfGFP (PDB 2b3p (Pedelacq et al., 2006)) as the freely rotating ligand. The models were ranked by the number of clustered simulations as calculated in balanced simulation mode (recognition of all intermolecular electrostatics), and the top ten ranked interfaces were downloaded as PDBs from the server for further analysis. Dynamic modelling of the molecular interfaces was further refined in ROSETTA’s high-resolution docking protocol (Liu and Kuhlman, 2006; Lyskov and Gray, 2008). The RosettaDock protocol ranked the predicted interfaces obtained within ClusPro simulation by both “Total Energy” and “Interface Energy”. Both the haem of cyt *b*_562_ and the chromophore of sfGFP were reintroduced back into each structure using PyMOL. The predicted heterodimer structure obtained by this method were therefore taken as the most energetically favourable and the top ranked model inspected in PyMOL for suitably close partner residues.

### Protein Engineering and Recombinant Production

The SCO-K (see Supplementary Figure S1A for chemical structure) containing sfGFP variants were generated and produced as described previously (Worthy et al., 2019; Pope et al., 2020). The gene encoding cyt *b*_562_ was present in the pBAD plasmid. The cyt *b*_562_^50AzF^ variant was generated and produced as described previously (Zaki et al., 2018; Thomas et al., 2020). The cyt *b*_562_^71AzF^ variant was generated by introducing a TAG codon in place of the Q71 encoding codon by whole plasmid PCR (Forward primer 5′-C GGT **TAG** ATT GAC GAC G-3′ and reverse primer 5′-AC CAG AAT GTC GAA ACC G-3). Incorporation of AzF (see Supplementary Figure S1A for chemical structure) into cyt *b*_562_ and its subsequent purification was achieved as described previously (Zaki et al., 2018; Thomas et al., 2020) using the pDULE plasmid (Miyake-Stoner et al., 2010). After cell lysis and removal of cellular debris, the soluble lysate was subjected to 30% (w/v) ammonium sulphate precipitation to remove some contaminant protein as precipitant. The soluble supernatant was subjected to a further round of ammonium sulphate precipitation to a concentration of 90% (w/v) to precipitate all protein. The precipitated lysate was resuspended 50 mM Tris pH 8.0 and if desired was mixed with 100x molar excess of haem porphyrin to generate of holo-cyt *b*_562_^71AzF^. Protein was applied to Hiload™ 16/600 Superdex™ S75. Fractions containing cyt *b*_562_ were applied to a Sepharose Q anion exchange column before final buffer exchange into 50 mM Tris pH 8.0 using a PD-10 desalting column. Pure cyt *b*_562_^71AzF^ was concentrated to 100 μM and separated into 100 μL samples before flash freezing and storage at −80°C until use. Apo-cyt *b*_562_^71AzF^ was generated by haem extraction essentially as described elsewhere (Jones and Barker, 2004; Bowen et al., 2020).

### Heterodimer Formation via SPAAC

Strain promoted azide-alkyne cycloaddition of protein was achieved by mixing of SCO-K containing sfGFP (50 µM) with cyt *b*_562_^71AzF^ (50 µM) at 37°C overnight. After incubation, the formation of oligomeric protein was determined by SDS-PAGE. Purification of oligomeric protein was achieved by size exclusion chromatography using Hiload™ 26/600 Superdex™ S200 gel filtration column (Section 2.4.2) and the purity of the resultant protein oligomer was assessed by SDS PAGE. Yield of dimer was estimated by ImageJ (Schindelin et al., 2012) analysis of band intensity of each form after Coomassie staining of polyacrylamide gels.

### Absorbance and Fluorescence Spectroscopy

UV-visible absorption spectra were recorded with Cary 60 spectrophotometer using 1 cm pathlength quartz cuvettes. Absorbance was recorded between 200–800 nm at a scan rate of 300 nm/min. The molar absorbance co-efficient (ε) for the sfGFP variants have been determined previously (Worthy et al., 2019). The concentration of cyt *b*_562_^71AzF^ was determined using the DC-protein assay (BioRad) using the wild-type cyt *b*_562_ as the standard. The subsequent molar absorbance co-efficient was calculated using the Beer-Lambert law with the absorbance of a known concentration of cyt *b*_562_^71AzF^. Fluorescence emission spectra were recorded on a Cary Eclipse Fluorimeter, using 5 mm × 5 mm QS quartz cuvette. Samples were excited at the λ_max_ and emission was recorded at every 1 nm from the point of excitation to 700 nm. A scan rate of 120 nm/min was used for all spectra recorded with a 5 nm slit width and voltage set to medium. To measure the emission in reducing conditions protein was first incubated with a 10-fold molar excess of DTT.

## Results

### *In Silico* Prediction of sfGFP-cyt *b*_562_ Interface

The first step in heterodimer design is to predict compatible interfaces between the two starting proteins. Previous work has shown that not all surface exposed residues are amenable to dimer formation via SPAAC (Worthy et al., 2019). We show the same appears true here. Incorporation of strained-cyclooctyne-lysine (SCO-K) in sfGFP at residue 204 is known to promote dimerisation with a mutually compatible interface (Worthy et al., 2019; Pope et al., 2020). Incorporation of azidophenylalanine (AzF) at residue 50 in cyt *b*_562_ places the non-canonical amino acid (ncAA) within a dynamic extended surface loop (Supplementary Figure S1B). Previous work has shown that cyt *b*_562_^50AzF^ is amenable to chemical functionalisation with non-biological entities (Zaki et al., 2018; Thomas et al., 2020). As shown in Supplementary Figure S1C, protein dimerisation did not occur to any great extent.

To predict potentially compatible interfaces between cyt *b*_562_ and sfGFP, we used an *in silico* docking approach developed recently for constructing fluorescent protein dimers (Worthy et al., 2019; Pope et al., 2020). The first step uses ClusPro (Kozakov et al., 2017) to generate unbiased docking of sfGFP (PDB 2b3p (Pedelacq et al., 2006)) to cyt *b*_562_ (1qpu (Arnesano et al., 1999)). Haem and the sfGFP chromophore are automatically removed leaving the core structures intact relative to the starting structure. Of the 30 alignments generated, the top 10 models ranked according to cluster number were further analysed (see Supplementary Figure S2 for alignments and cluster information). None included an interface involving residue 50 in cyt *b*_562_. To provide a more quantitative analysis and refine the docking procedure, each initial model was further assessed and ranked using RosettaDock (Leaver-Fay et al., 2011; Alford et al., 2017) through the generation of an estimated energy of the interface and total energy between the molecules. The top five models are shown in Figure 1B with the energies in Supplementary Table S1. The distances between the two chromophores varied from 20.5 Å in the highest ranked model (RD1) to 32.4 Å. While model RD9 had the marginally lowest interface energy, RD1 had the lowest total energy and was derived from the largest cluster number (CP1 in Supplementary Figure S1). Thus, RD1 was taken forward as the primary model to base the design of SPAAC linkage sites.

Analysis of RD1 revealed that two previous residues in sfGFP known to successfully promote dimerisation via SPAAC, 148 and 204 (Worthy et al., 2019), were located close to the dimer interface (Figure 1C). Residue H148 is critical to sfGFP function as it helps define the fluorescent properties through H-bonding to the chromophore’s phenolic group. Changing H148 to a ncAA is tolerated and changes the inherent fluorescence properties (Reddington et al., 2013; Hartley et al., 2016; Worthy et al., 2019). Q204 to is also tolerant to ncAA incorporation (Reddington et al., 2012; Worthy et al., 2019). Thus, these residues were selected within the context of sfGFP. In cyt *b*_562_ helices three and four comprised the main docking interface; residue Q71 in helix three was chosen as it was close to both residues 148 and 204 in sfGFP (Figure 1C). The SCO-K ncAA was previously incorporated into sfGFP residues 148 (sfGFP^148SCO^) and 204 (sfGFP^204SCO^) and characterised (Worthy et al., 2019). Incorporation of azF into cyt *b*_562_ at residue 71 (cyt *b*_562_^71azF^) in response to a TAG codon has been demonstrated previously as part of a separate directed evolution codon exchange study (Arpino et al., 2015). Cyt *b*_562_^71azF^ produced here has similar spectral characteristic to that of wild-type cyt *b*_562_ (Supplementary Figure S3).

### Cycloaddition of sfGFP and Cyt *b*_562_ and Its Impact of Absorbance

**FIGURE 2:**
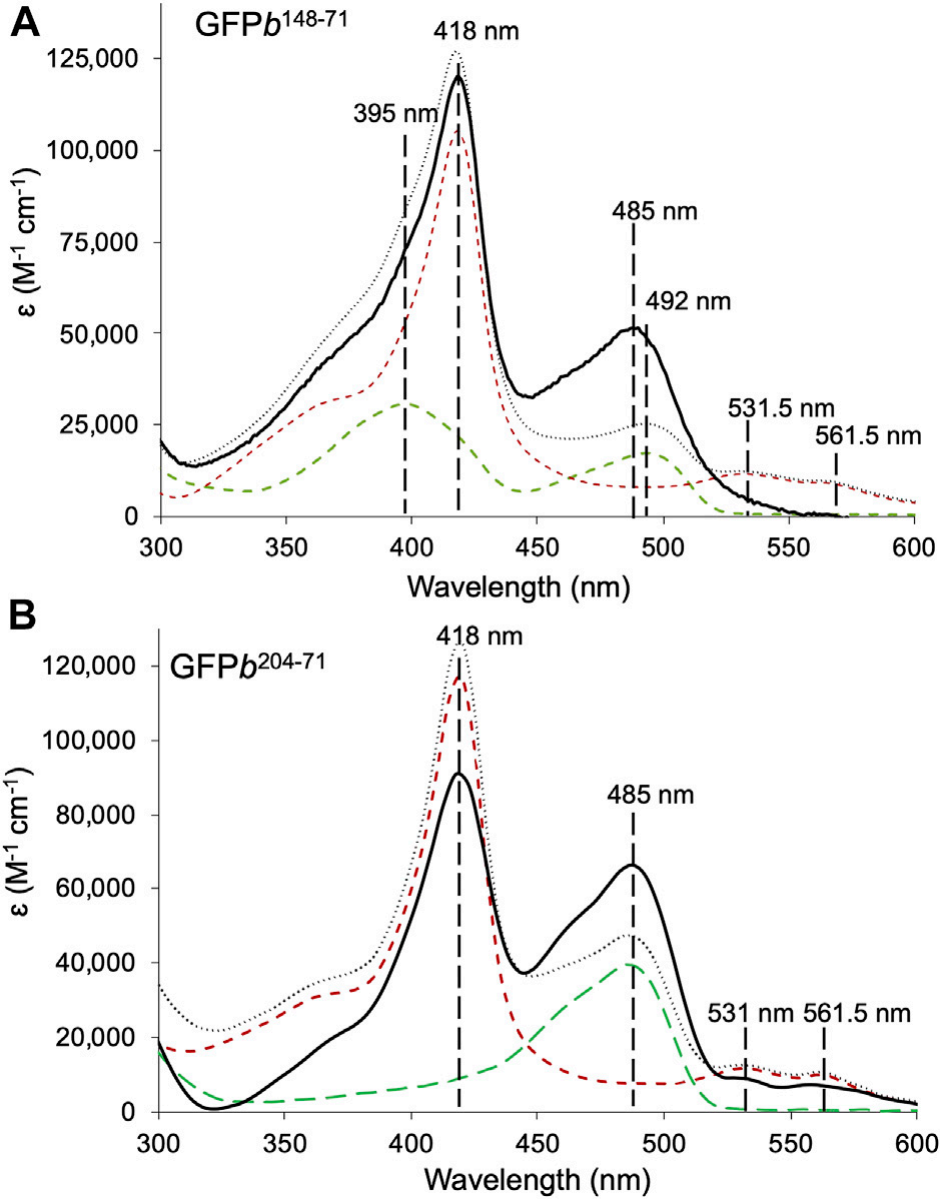
Absorbance spectra of sfGFP-cyt *b*_562_ heterodimers. **(A)** GFP*b* ^148-71^ (black solid line), and constituent monomers sfGFP^148SCO^ (green dashed line) and cyt *b*_562_^71azF^ (red dashed line). **(B)** GFP*b* ^204-71^ (black solid line), and constituent monomers sfGFP^204SCO^ (green dashed line) and cyt *b*_562_^71azF^. The combined sum of each monomeric absorbance is shown for comparison (grey dotted line). Data shown as molar extinction coefficients. The wavelengths associated with the λ_max_ for each monomer absorbance peak is shown for reference. The molar absorbance coefficients are shown in Supplementary Table S2.

Analysis by SDS-PAGE revealed that dimerisation of cyt *b*_562_^71azF^ with sfGFP^148SCO^ or sfGFP^204SCO^ were successful, with yields in the range of 20–35% (see Supplementary Figure S4 for representative SDS-PAGE gels). The yields are slightly lower compared SPAAC based dimerisation of structurally similar proteins (35–80%) (Worthy et al., 2019). The two new heterodimers termed GFP*b* ^148-71^ and GFP*b* ^204-71^ were isolated from their monomeric forms by size exclusion chromatography. The absorbance spectra have characteristics of both constituent proteins with major peaks at 418 nm equivalent to cyt *b*_562_ and ∼485 nm contributed by sfGFP (Figure 2). The absorbance spectra of the heterodimers also indicate positive functional changes on dimerisation with respect to the sfGFP unit (Figure 2 with molar extinctions provided in Supplementary Table S2).

In terms of GFP*b* ^148-71^, compared to the starting monomers, the absorbance peak at ∼485 nm associated with sfGFP^148SCO^ increases by just over 3-fold in the heterodimer with a concomitant drop in the shoulder at 395 nm (Figure 2A). Such spectral characteristics are associated with a switch in the protonation state of sfGFP chromophore; the neutral phenol chromophore has a peak absorbance of ∼400 nm and the phenolate anionic form absorbs at ∼485 nm (Remington, 2011). Simple addition of the monomer absorbance spectra confirms the promotion of the anionic sfGFP chromophore form rather than any baseline addition from the cyt *b*_562_ unit. Promotion of the anionic chromophore has been shown previously for symmetrically arranged sfGFP homodimers linked by residue 148 (Worthy et al., 2019). Thus, modulation of the sfGFP chromophore charged state and hence function is still feasible when linked to a very distinct partner protein.

GFP*b* ^204-71^ also shows a significant increase in absorbance corresponding the sfGFP chromophore, with molar absorbance at 485 nm almost doubling (Figure 2B). The simple monomer addition spectrum confirms that the increase in the 485 nm absorbance is not due to an underlying contribution by cyt *b*_562_. Thus, the ability of sfGFP to interact with light has been enhanced on dimerisation. Unlike sfGFP^148SCO^, sfGFP^204SCO^ exists predominantly in the anionic state so the increase in 485 nm absorbance is not down to change in chromophore ionisation state. We have previously proposed that such a positive synergistic effect is due to reduced water dynamics in channels leading to the sfGFP chromophore when homo-dimerisation occurs via residue 204 (Pope et al., 2020); the same may also be occurring here.

### Functional Communication in the Heterodimers

**FIGURE 3:**
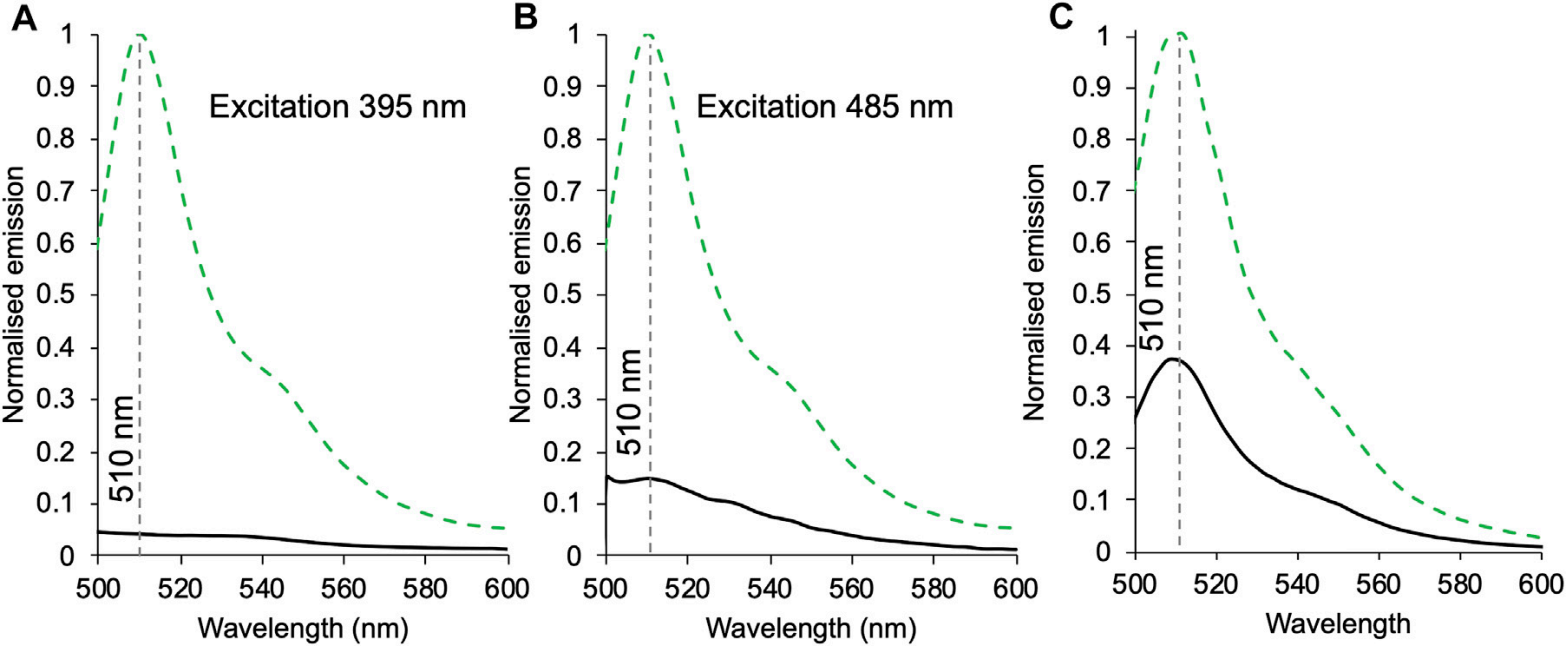
The effect of heterodimerisation on fluorescence emission. Emission of 1 μM of sfGFP^148SCO^ (green dashed) or GFP*b* ^148-71^ (black) excited at either 395 nm **(A)** or 485 nm **(B)**. **(C)** Emission of 1 μM sfGFP^204SCO^ (green dashed) and GFP*b* ^204-71^ (black) on excitation at 485 nm. Spectra were normalised to sfGFP^148SCO^ **(A, B)** or sfGFP^204SCO^ **(C)**.

Haem can quench fluorescence by resonance energy transfer, providing the fluorophore is within close proximity (Willis et al., 1990; Takeda et al., 2001; Arpino et al., 2012). The requirement for close proximity is shown in Supplementary Figure S5, where free haem or free cyt *b*_562_ do not quench sfGFP to any appreciable extent. To assess communication between sfGFP and cyt *b*_562_ in our heterodimers, fluorescence was measured on excitation at the major absorbance peaks of sfGFP (Figure 3). As GFP*b* ^148-71^ has two potential absorbance peaks, excitation was performed at both 395 and 485 nm (Figures 3A,B). On excitation at 395 nm, emission is reduced by 96% compared to the monomer, and on excitation at 485 nm emission was reduced by 85%. The difference between the two may be due to the relative change in absorbance at each wavelength on conversion from a monomer to heterodimer; cyt *b*_562_ may also absorb some of the 395 nm light (see Figure 2A for spectral overlap). This still represents a major reduction in fluorescence suggesting a high degree of energy transfer and thus communication between sfGFP and cyt *b*_562_ in the GFP*b* ^148-71^ construct. By comparison, the drop in emission on excitation of GFP*b* ^204-71^ is 67% (Figure 3C). This suggests that while energy transfer is still occurring the efficiency is reduced compared to GFP*b* ^148-71^. SEC clearly resolves the heterodimer from the monomers (Supplementary Figure S6) thus we do not believe contaminating monomeric sfGFP^204SOC^ is the cause of the residual fluorescence observed for GFP*b* ^204-71^.

### Haem and Redox-State Dependent Quenching in GFP*b* ^204-71^

**FIGURE 4:**
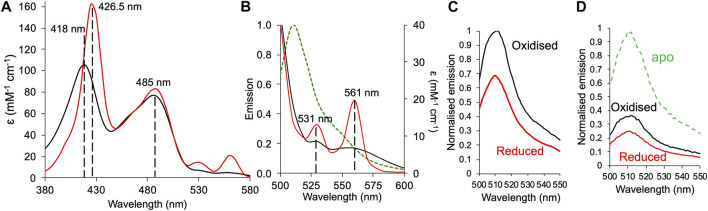
Redox dependent fluorescence emission of GFP*b* ^204-71^. **(A)** Absorbance spectra of GFP*b* ^204-71^ under oxidising (black line) and reduced (red line). Reducing conditions stimulated by addition of a 10-fold molar excess of DTT. **(B)** Absorbance spectra under oxidising and reducing conditions shown in **(A)** for the α/β peak region overlaid with the emission spectrum (on excitation at 485 nm) of sfGFP (green dashed line). **(C)** Emission spectra of 1 μM GFP*b* ^204-71^ in the absence (oxidised; black) and presence (reduced; red) of DTT. Spectra were normalised to oxidised holo-GFP*b* ^204-71^. **(D)** The emission spectra of apo-GFP*b* ^204-71^ before the addition of haem under oxidising (black line) or reduced (red line) conditions. Spectra were normalised to apo-GFP*b* ^204-71^.

The iron centre of the cyt *b*_562_ haem group switches between the reduced Fe^2+^ and oxidised Fe^3+^ state that results in changes to the absorbance spectrum and affinity for the protein (Robinson et al., 1997; Wittung-Stafshede et al., 1999; Jones and Barker, 2005). The observed fluorescence emission from GFP*b* ^204-71^ allows us to monitor how fluorescence output can be tuned to both haem binding and redox conditions. Conversion from oxidised to reduced haem was achieved through the addition of the reducing agent dithiothreitol (DTT). The cyt *b*_562_ unit in GFP*b* ^204-71^ is still capable of redox state switching as shown by the switch in the 418 nm absorbance peak for the oxidised form to 426 nm characteristic of reduced cyt *b*_562_ with the typical higher molar absorbance (Figure 4A and Supplementary Table S3). The α/β band peaks also become more prominent as expected on conversion from oxidised to reduced cyt *b*_562_, which in turn increases the spectral overlap between the sfGFP emission and cyt *b*_562_ absorbance (Figure 4B). As anticipated, the reducing agent had little impact on sfGFP absorbance (Figure 4A). On the addition of reducing agent, fluorescence emission dropped by an additional 31% (Figure 4C). Thus, GFP*b* ^204-71^ output can respond to changes in redox conditions through coupling changes in the redox state of haem iron bound to cyt *b*_562_ to fluorescence output of sfGFP.

We thus attempted to extend this to see if the apo-heterodimer (no haem bound) could bind and respond to haem. Linking haem binding to cyt *b*_562_ with fluorescent protein output are potentially useful biosensors for this important biological co-factor (Takeda et al., 2003; Arpino et al., 2012; Hanna et al., 2016). The apo-GFP*b* ^204-71^ responded to haem binding and subsequent switch in redox conditions. On addition of haem to apo-GF*b* ^204-71^, fluorescence emission dropped by 64% and by a further 12% (equivalent to a 33% compared to the oxidised holo-GF*b* ^204-41^) on addition of reducing agent (Figure 4D). These results are comparable to the results observed above for holo-GF*b* ^204-41^ (when compared to monomeric GFP^204SCO^; Figure 3C) suggesting under the conditions used, full haem binding has occurred to the apo-protein, and the heterodimer responds to change in redox conditions.

## Discussion

While traditionally protein engineering has focused on converting oligomeric proteins into monomers, especially with regards to fluorescent proteins (Zacharias et al., 2002; Scott et al., 2018), the design and construction of artificial protein oligomers or “supramolecular” systems is currently of great interest (Norn and Andre, 2016; Yeates et al., 2016; Kobayashi and Arai, 2017; Beesley and Woolfson, 2019; Gwyther et al., 2019). Both GFP (Ding et al., 2015; Kim et al., 2015; Leibly et al., 2015; Worthy et al., 2019; Arpino and Polizzi, 2020) and cyt *b*_562_ (Baldwin et al., 2006; Radford et al., 2011; Brodin et al., 2012; Onoda et al., 2012; Song and Tezcan, 2014; Oohora et al., 2018; Golub et al., 2020) have been central to exemplifying the ability to generate new oligomeric supramolecular protein structures (Oohora and Hayashi, 2014). Artificial protein oligomers offer the same potential impact as oligomerisation does in nature: the generation of complex higher-order structures from a limited monomeric building block repertoire. This in turn allows new structural and thus functional space to be sampled not accessible in monomeric proteins. Whether it is O_2_ binding to haemoglobin (Ciaccio et al., 2008) or enzyme catalysis and allosteric regulation (Goodsell and Olson, 2000; Ali and Imperiali, 2005), one of the main benefits of oligomerisation is synergy between individual subunits. Such linked functionality is generally hard to design and engineer into artificial complexes due to the requirement of long-range interactions that link active sites. Thus, most designed protein complexes focus on the interface region. We have shown previously that such networks can be generated between fluorescent protein homo and heterodimers using an approach like that used here, which leads to functional switching and fluorescence enhancement (Worthy et al., 2019).

Here we decided to test our ability to select compatible interfaces between disparate proteins by choosing two monomeric proteins with very different structures: a largely β-sheet protein (sfGFP) and a helical protein (cyt *b*_562_). The rationale for using SPAAC to covalently stabilise the heterodimer structure is that classical approaches such as disulphide bridges cannot discriminate between homo-dimers and heterodimers leading to a mixed population; the bioorthogonal nature of SPAAC means only heterodimer will form. Furthermore, the ncAAs used here have longer side chains than the short -CH_2_-SH group of cysteine meaning steric clashes between monomers is less likely to inhibit covalent bond formation but still allow an intimate interaction between the individual monomer units. The triazole link is also more stable than a disulphide bridge. While our aim was not to generate a newly designed dimer interface mimicking natural protein-protein interactions, we did need to identify compatible interfaces that will at least persist for a length of time to allow covalent functionalisation. Without identifying compatible interfaces, the chances of stabilising the interface through SPAAC is minimal. Residue 50 in cyt *b*_562_ is largely surface exposed in a flexible extended loop and would normally be considered an ideal residue to target for covalent coupling with another protein (Supplementary Figure S1B) but SPAAC facilitated dimerisation was not possible via this residue (Supplementary Figure S1C). Previous work has shown that incorporation of azF at residue 50 is reactive (Zaki et al., 2018; Thomas et al., 2020). Lack of dimerisation is thus likely a result of the individual subunits unable to become spatially localised for long enough to promote SPAAC. This mirrors previous work with fluorescent proteins that suggested compatible interfaces are required for SPAAC facilitate dimerisation even when surface exposed residues are selected (Worthy et al., 2019)). Our computational design approach proved successful both here (Figure 1 and Supplementary Figure S4) and elsewhere (Worthy et al., 2019; Pope et al., 2020) in identifying compatible interfaces. It also provides a rationale for the inability of cyt *b*_562_^50azF^ to form dimers with sfGFP as residue 50 is not involved in any of the predicted protein interfaces. The step forward here was using proteins with disparate structural folds. The overall total binding energy was higher here (less negative) than for structurally homologues proteins tested previously suggesting a weaker interaction (Supplementary Figure S2 and Supplementary Table S2 *versus* data in references (Worthy et al., 2019; Pope et al., 2020)) but still proved useful in generating models for predicting successful coupling sites (Figure 1). The model suggests that there are intermolecular interactions between cyt *b*_562_ and sfGFP beyond residues involved in SPAAC, but it is not clear if they persist in the SPAAC link dimer. However, our previous structural work with SPAAC linked fluorescent proteins dimers shows that extensive intermolecular non-covalent interactions are formed at the interface, including long range interaction networks linking the active sites (Worthy et al., 2019; Pope et al., 2020). Thus, given the intimate nature of the coupling between sfGFP and cyt *b*_562_, additional non-covalent interactions outside the triazole linkage between the two monomers on forming the dimer are highly likely to be present.

Two heterodimers were designed based on the top ranked model (Figure 1C). In terms of sfGFP, residues 148 and 204 were selected to host the strained alkyne ncAA (SCO-K). These sites have been shown previously to be amenable to both small molecule (Reddington et al., 2012; Hartley et al., 2016) and protein (Worthy et al., 2019; Pope et al., 2020) attachment via SPAAC. H148 in sfGFP forms a H-bond with the chromophore, which directly impacts on the fluorescence properties of the protein by assisting in deprotonation of the chromophore’s phenol group. In many crystal structures, H148’s side chain is largely buried but is known to be dynamic (Seifert et al., 2003) with the “flipped out” conformation observed in some crystal structure populations, resulting in the residue becoming solvent exposed (Reddington et al., 2013; Arpino et al., 2014). Changing H148 to a larger ncAA results in breakage of a critical H-bond with the chromophore and exclusion from the core of the protein due to steric clashes (Hartley et al., 2016); the residue now amenable to chemical modification. The result is a mixed population of the protonated and anionic form of the sfGFP chromophore prior to SPAAC modification (Figure 2A). We have previously shown that we can control the relative populations of two chromophore states causing switching of the fluorescent properties in terms of excitation wavelength (Reddington et al., 2013; Hartley et al., 2016; Worthy et al., 2019). We have successfully achieved the same here through the formation of a heterodimer with promotion of the anionic form on dimerisation (Figure 2A). As mutation of H148 to a ncAA removes the H-bond to the sfGFP chromophore critical to formation of the phenolic anion, we have proposed previously that a structural water molecule replaces the imidazole group and plays the role of the H-bond acceptor that promotes ionisation of the chromophore (Worthy et al., 2019; Pope et al., 2020); the same scenario may also be the case here with a water molecule trapped at the sfGFP-*b*_562_ interface in a position to H-bond to the chromophore.

With respect to GFP*b* ^204-71^, we see enhancement of the sfGFP molar absorbance (Figure 2B). Unlike H148, Q204 is surface exposed and plays little role in dictating the fluorescence properties of sfGFP, even when replaced by an ncAA (Reddington et al., 2012; Worthy et al., 2019). Water dynamics is again thought to play a major role. Dimerisation of sfGFP with itself or closely related fluorescent proteins enhances molar absorbance and structural analysis revealed ordered water molecules at the dimer interface, including waters comprising a channel through to the chromophore (Pope et al., 2020). The same may be happening with GFP*b* ^204-71^ with the dimer interface trapping water molecules leading to a sustained water-protein bond network that improves the ability of the sfGFP chromophore to interact with light.

**FIGURE 5:**
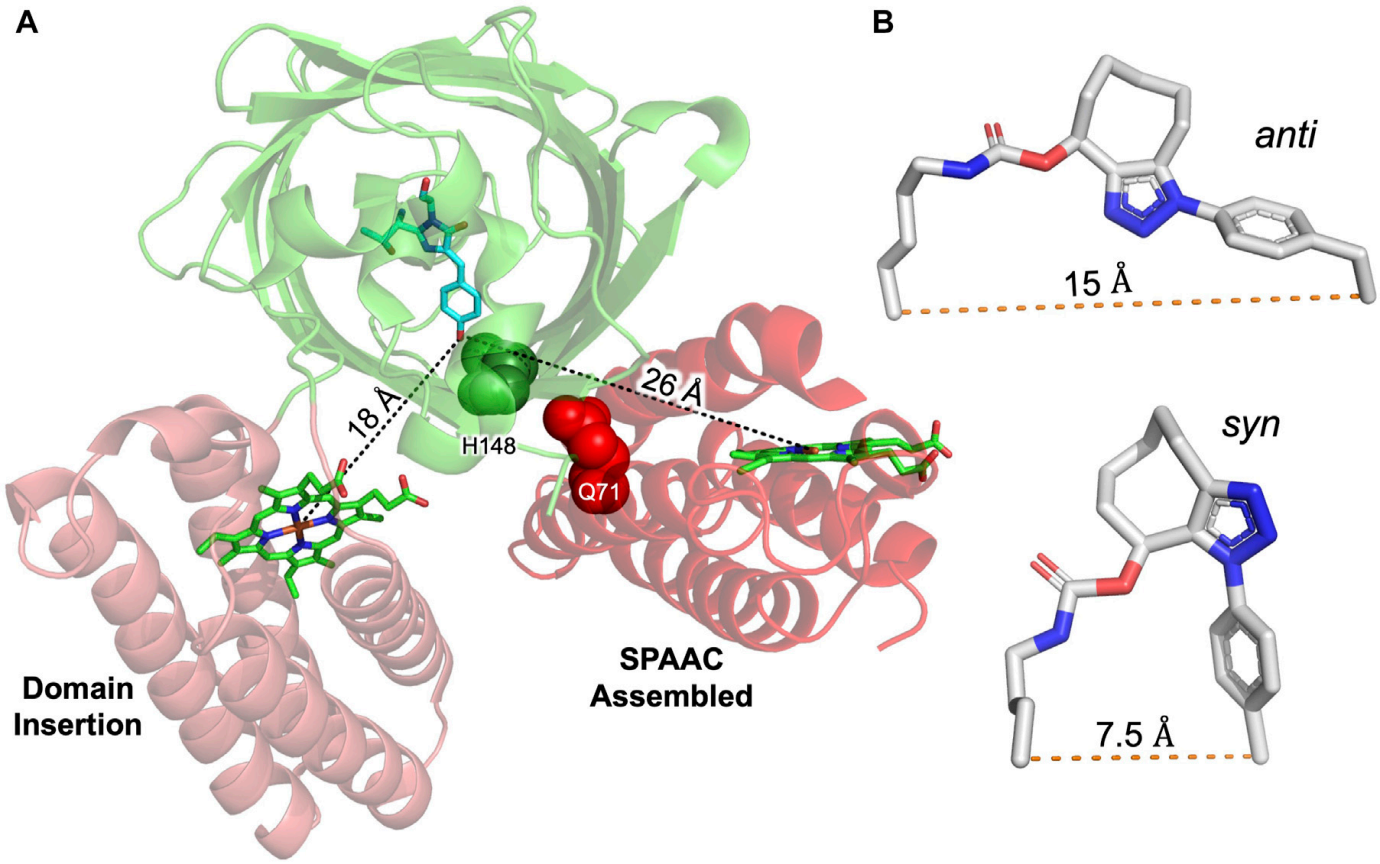
Comparison of chromophore distances between GFP and cyt *b*_562_ constructed by domain insertion or SPAAC assembly. Alignment of crystal structure (3u8p) of EGFP-cyt *b*_562_ CG6 domain insert variant (Arpino et al., 2012) with the highest ranked model of sfGFP (2b3p) and cyt *b*_562_ (1 qpu) obtained by RosettaDock. GFP (green) used as centre of alignment with chromophore shown (cyan sticks). The measured distance between the GFP chromophore phenolic hydroxyl group and central iron of each haem chromophore are shown associated with the black dotted lines. Residues mutated to ncAAs are shown as spheres and labelled. **(B)** The *anti* (derived from PDB 5 nhn (Worthy et al., 2019))and *syn* (derived from PDB 5ni3 (Pope et al., 2020)) configurations around the triazole bond with the relative distances between the C_α_ of the two original amino acids shown.

Bringing the sfGFP chromophore within close proximity to haem through formation of the heterodimer should result in energy transfer from sfGFP that is quenched by cyt *b*_562_, a feature that has been observed before for single polypeptide systems (Takeda et al., 2003; Arpino et al., 2012; Hanna et al., 2016). The extent of quenching is related to the distance between the two chromophores (Arpino et al., 2012). Quenching in a classical N- or C-terminal fusion of EGFP to cyt *b*_562_ was less than 65% (Arpino et al., 2012). Functional communication was present in both our heterodimers, but the efficiency of energy transfer differed depending on the SPAAC linkage positions, haem redox state and, to an extent, the excitation wavelength (Figure 3). This is despite the two residues being adjacent to each other in the structure. Linkage via sfGFP residue 148 had the highest energy transfer efficiency (>85%) with more apparent efficient energy transfer on excitation 395 nm. Linkage via sfGFP residue 204 resulted in a lower energy transfer (63%), comparable to previous “head-to-tail” fusions with cyt *b*_562_ where the two chromophores are not anticipated to be close in space (Arpino et al., 2012). Thus, the question arises as to why different linkage sites that are close together in sfGFP (Figure 1C) generate very different energy transfer efficiencies? Given that GFP*b* ^204-71^ can be separated from its monomeric components by size exclusion chromatography (Supplementary Figure S6), the residual fluorescence is unlikely to be from contaminating sfGFP^204SCO^ monomer. We have shown previously that linkage via residue 204 in fluorescent protein dimers resulted in lower than predicted energy transfer (Pope et al., 2020). The exact reason was not clear. The calculated *R*_0_ (the Förster radius at which energy transfer is 50% efficient) between EGFP and cyt *b*_562_ is 46 Å (Takeda et al., 2001). As energy transfer is related to the donor-acceptor chromophore distance (*r*) and energy transfer (*E*) through equation *E* = 1/[1+(r/*R*_0_)^6^], we can estimate the distance between the chromophores. Previously, we showed that there was a good correlation between *r* from energy transfer (99%) and structure in our domain insert EGFP-cyt *b*_562_ construct (Figure 5). Based on energy transfer efficiency, in GFP*b* ^148-71^ the closest interchromophore distance is predicted to be ∼27 Å while for GFP*b* ^204-71^ it is ∼41 Å. The interchromophore distances derived from the original top ranked model suggest that it might provide a realistic representation for GFP*b* ^148-71^ but not GFP*b* ^204-71^ (Figure 5). As shown in Figure 5, the domain arrangement in our heterodimer model compared to the determined structure of the high energy transfer efficient domain insert protein is different; the interchromophore distance is 8 Å longer thus could account for the slightly reduced energy transfer efficiency in GFP*b* ^148-71^. Residue 204 is close to the interface rather than directly forming the interface, which may result in the two monomers adjusting their relative placement on SPAAC compared to that of the model. There are two isomeric forms for the triazole crosslink between AzF and SCO-K (Worthy et al., 2019; Pope et al., 2020): the *syn* isomer that forms a turn structure and the *anti* form that results in an elongated linkage (Figure 5B). The *anti* form may dominate for GFP*b* ^204-71^ resulting in the two monomers and thus chromophores being separated by a longer distance. Interestingly, energy transfer efficiency was oxidation state-dependent (Figures 4C,D). The ability apo-GFP*b* ^204-71^ to bind haem suggests that the haem binding site is still accessible as predicted by our original modelling and on binding haem under non-reducing conditions exhibits a similar drop in fluorescence to that observed for holo-GFP*b* ^204-71^ suggesting full haem occupancy (Figures 3C, 4D). On addition of reducing agent, fluorescence decreased by another third suggesting conversion from Fe^3+^ to Fe^2+^ resulted in increased energy transfer efficiency. DTT is not known to reduce the fluorescence emission spectra of sfGFP (Supplementary Figure S7 and (Reddington et al., 2015)) so the increased energy transfer efficiency may due to inherent change in the protein-bound haem such as increased molar absorbance of the α/β peaks that overlap with the sfGFP emission (Figure 4B). Even under reducing conditions, energy transfer efficiency is still ∼75% equating to an interchromophore distance of 38 Å. Thus, we cannot rule out other currently unknown events contributing to the reduced energy transfer efficiency in GFP*b* ^204-71^, as also observed in fluorescent protein dimers linked via residue 204 (Pope et al., 2020).

To conclude, here we have shown that it is feasible to design and construct artificial heterodimers between structurally and functionally disparate proteins linked by a genetically encoded bio-orthogonal link. The *in silico* docking helped identify suitability compatible protein-protein interfaces that where then stabilised by a triazole link formed by SPAAC. In both heterodimer configurations tested, the ability of phenol anion chromophore form of the sfGFP to interact with light was enhanced, with energy transfer to the haem centre of cyt *b*_562_ demonstrating functional linkage.

## Data Availability

The datasets presented in this study can be found in online repositories. The names of the repository/repositories and accession number(s) can be found below: http://doi.org/10.17035/d.2021.0080088158.

## References

[B1] Alford R. F. Leaver-Fay A. Jeliazkov J. R. O’Meara M. J. Dimaio F. P. Park H. (2017). The Rosetta All-Atom Energy Function for Macromolecular Modeling and Design. J. Chem. Theor. Comput. 13, 3031–3048. 10.1021/acs.jctc.7b00125 PMC5717763 28430426

[B2] Ali M. H. Imperiali B. (2005). Protein Oligomerization: How and Why. Bioorg. Med. Chem. 13, 5013–5020. 10.1016/j.bmc.2005.05.037 15993087

[B3] Arnesano F. Banci L. Bertini I. Faraone-Mennella J. Rosato A. Barker P. D. (1999). The Solution Structure of Oxidized Escherichia coli Cytochrome B562,. Biochemistry 38, 8657–8670. 10.1021/bi982785f 10393541

[B4] Arpino J. A. Baldwin A. J. Mcgarrity A. R. Tippmann E. M. Jones D. D. (2015). In-frame Amber Stop Codon Replacement Mutagenesis for the Directed Evolution of Proteins Containing Non-canonical Amino Acids: Identification of Residues Open to Bio-Orthogonal Modification. PLoS One 10, e0127504. 10.1371/journal.pone.0127504 26011713 PMC4444182

[B5] Arpino J. A. J. Czapinska H. Piasecka A. Edwards W. R. Barker P. Gajda M. J. (2012). Structural Basis for Efficient Chromophore Communication and Energy Transfer in a Constructed Didomain Protein Scaffold. J. Am. Chem. Soc. 134, 13632–13640. 10.1021/ja301987h 22822710

[B6] Arpino J. A. J. Polizzi K. M. (2020). A Modular Method for Directing Protein Self-Assembly. ACS Synth. Biol. 9, 993–1002. 10.1021/acssynbio.9b00504 32243747

[B7] Arpino J. A. J. Rizkallah P. J. Jones D. D. (2014). Structural and Dynamic Changes Associated with Beneficial Engineered Single-Amino-Acid Deletion Mutations in Enhanced green Fluorescent Protein. Acta Cryst. D Biol. Crystallogr. 70, 2152–2162. 10.1107/s139900471401267x 25084334 PMC4118826

[B8] Baldwin A. J. Bader R. Christodoulou J. Macphee C. E. Dobson C. M. Barker P. D. (2006). Cytochrome Display on Amyloid Fibrils. J. Am. Chem. Soc. 128, 2162–2163. 10.1021/ja0565673 16478140

[B9] Beesley J. L. Woolfson D. N. (2019). The De Novo Design of α-helical Peptides for Supramolecular Self-Assembly. Curr. Opin. Biotechnol. 58, 175–182. 10.1016/j.copbio.2019.03.017 31039508

[B10] Bowen B. J. Mcgarrity A. R. Szeto J.-Y. A. Pudney C. R. Jones D. D. (2020). Switching Protein Metalloporphyrin Binding Specificity by Design from Iron to Fluorogenic Zinc. Chem. Commun. 56, 4308–4311. 10.1039/d0cc00596g 32186552

[B11] Brodin J. D. Ambroggio X. I. Tang C. Parent K. N. Baker T. S. Tezcan F. A. (2012). Metal-directed, Chemically Tunable Assembly of One-, Two- and Three-Dimensional Crystalline Protein Arrays. Nat. Chem 4, 375–382. 10.1038/nchem.1290 22522257 PMC3335442

[B12] Butterfield G. L. Lajoie M. J. Gustafson H. H. Sellers D. L. Nattermann U. Ellis D. (2017). Evolution of a Designed Protein Assembly Encapsulating its Own RNA Genome. Nature 552, 415–420. 10.1038/nature25157 29236688 PMC5927965

[B13] Ciaccio C. Coletta A. De Sanctis G. Marini S. Coletta M. (2008). Cooperativity and Allostery in Haemoglobin Function. IUBMB Life 60, 112–123. 10.1002/iub.6 18380000

[B14] Ding Y. Li J. Enterina J. R. Shen Y. Zhang I. Tewson P. H. (2015). Ratiometric Biosensors Based on Dimerization-dependent Fluorescent Protein Exchange. Nat. Methods 12, 195–198. 10.1038/nmeth.3261 25622108 PMC4344385

[B15] Edwards W. R. Busse K. Allemann R. K. Jones D. D. (2008). Linking the Functions of Unrelated Proteins Using a Novel Directed Evolution Domain Insertion Method. Nucleic Acids Res. 36, e78. 10.1093/nar/gkn363 18559359 PMC2490766

[B16] Eger S. Scheffner M. Marx A. Rubini M. (2010). Synthesis of Defined Ubiquitin Dimers. J. Am. Chem. Soc. 132, 16337–16339. 10.1021/ja1072838 21033666

[B17] Fallas J. A. Ueda G. Sheffler W. Nguyen V. Mcnamara D. E. Sankaran B. (2017). Computational Design of Self-Assembling Cyclic Protein Homo-Oligomers. Nat. Chem. 9, 353–360. 10.1038/nchem.2673 28338692 PMC5367466

[B18] Golub E. Subramanian R. H. Esselborn J. Alberstein R. G. Bailey J. B. Chiong J. A. (2020). Constructing Protein Polyhedra via Orthogonal Chemical Interactions. Nature 578, 172–176. 10.1038/s41586-019-1928-2 31969701 PMC7007351

[B19] Gonen S. Dimaio F. Gonen T. Baker D. (2015). Design of Ordered Two-Dimensional Arrays Mediated by Noncovalent Protein-Protein Interfaces. Science 348, 1365–1368. 10.1126/science.aaa9897 26089516

[B20] Goodsell D. S. Olson A. J. (2000). Structural Symmetry and Protein Function. Annu. Rev. Biophys. Biomol. Struct. 29, 105–153. 10.1146/annurev.biophys.29.1.105 10940245

[B21] Gwyther R. E. A. Jones D. D. Worthy H. L. (2019). Better Together: Building Protein Oligomers Naturally and by Design. Biochem. Soc. Trans. 47, 1773–1780. 10.1042/bst20190283 31803901 PMC6925524

[B22] Hanna D. A. Harvey R. M. Martinez-Guzman O. Yuan X. Chandrasekharan B. Raju G. (2016). Heme Dynamics and Trafficking Factors Revealed by Genetically Encoded Fluorescent Heme Sensors. Proc. Natl. Acad. Sci. USA 113, 7539–7544. 10.1073/pnas.1523802113 27247412 PMC4941510

[B23] Hartley A. M. Worthy H. L. Reddington S. C. Rizkallah P. J. Jones D. D. (2016). Molecular Basis for Functional Switching of GFP by Two Disparate Non-native post-translational Modifications of a Phenyl Azide Reaction Handle. Chem. Sci. 7, 6484–6491. 10.1039/c6sc00944a 28451106 PMC5355941

[B24] Hatzakis N. S. Engelkamp H. Velonia K. Hofkens J. Christianen P. C. Svendsen A. (2006). Synthesis and Single Enzyme Activity of a Clicked Lipase-BSA Hetero-Dimer. Chem. Commun. 19, 2012–2014. 10.1039/B516551B 16767259

[B25] Hudak J. E. Barfield R. M. de Hart G. W. Grob P. Nogales E. Bertozzi C. R. (2012). Synthesis of Heterobifunctional Protein Fusions Using Copper-free Click Chemistry and the Aldehyde Tag. Angew. Chem. Int. Ed. 51, 4161–4165. 10.1002/anie.201108130 PMC3379715 22407566

[B26] Jones D. D. Barker P. D. (2005). Controlling Self-Assembly by Linking Protein Folding, DNA Binding, and the Redox Chemistry of Heme. Angew. Chem. Int. Ed. 44, 6337–6341. 10.1002/anie.200463035 16163771

[B27] Jones D. D. Barker P. D. (2004). Design and Characterisation of an Artificial DNA-Binding Cytochrome. Chembiochem 5, 964–971. 10.1002/cbic.200300569 15239054

[B28] Kim C. H. Axup J. Y. Dubrovska A. Kazane S. A. Hutchins B. A. Wold E. D. (2012). Synthesis of Bispecific Antibodies Using Genetically Encoded Unnatural Amino Acids. J. Am. Chem. Soc. 134, 9918–9921. 10.1021/ja303904e 22642368 PMC4299457

[B29] Kim S. Ko W. Sung B. H. Kim S. C. Lee H. S. (2016). Direct Protein-Protein Conjugation by Genetically Introducing Bioorthogonal Functional Groups into Proteins. Bioorg. Med. Chem. 24, 5816–5822. 10.1016/j.bmc.2016.09.035 27670101

[B30] Kim Y. E. Kim Y. N. Kim J. A. Kim H. M. Jung Y. (2015). Green Fluorescent Protein Nanopolygons as Monodisperse Supramolecular Assemblies of Functional Proteins with Defined Valency. Nat. Commun. 6, 7134. 10.1038/ncomms8134 25972078 PMC4479010

[B31] Kobayashi N. Arai R. (2017). Design and Construction of Self-Assembling Supramolecular Protein Complexes Using Artificial and Fusion Proteins as Nanoscale Building Blocks. Curr. Opin. Biotechnol. 46, 57–65. 10.1016/j.copbio.2017.01.001 28160725

[B32] Kozakov D. Hall D. R. Xia B. Porter K. A. Padhorny D. Yueh C. (2017). The ClusPro Web Server for Protein-Protein Docking. Nat. Protoc. 12, 255–278. 10.1038/nprot.2016.169 28079879 PMC5540229

[B33] Larsen T. A. Olson A. J. Goodsell D. S. (1998). Morphology of Protein-Protein Interfaces. Structure 6, 421–427. 10.1016/s0969-2126(98)00044-6 9562553

[B34] Leaver-Fay A. Tyka M. Lewis S. M. Lange O. F. Thompson J. Jacak R. (2011). Rosetta3. Methods Enzymol. 487, 545–574. 10.1016/b978-0-12-381270-4.00019-6 21187238 PMC4083816

[B35] Leibly D. J. Arbing M. A. Pashkov I. Devore N. Waldo G. S. Terwilliger T. C. (2015). A Suite of Engineered GFP Molecules for Oligomeric Scaffolding. Structure 23, 1754–1768. 10.1016/j.str.2015.07.008 26278175 PMC4568552

[B36] Liu Y. Kuhlman B. (2006). RosettaDesign Server for Protein Design. Nucleic Acids Res. 34, W235–W238. 10.1093/nar/gkl163 16845000 PMC1538902

[B37] Ljubetic A. Gradisar H. Jerala R. (2017). Advances in Design of Protein Folds and Assemblies. Curr. Opin. Chem. Biol. 40, 65–71. 10.1016/j.cbpa.2017.06.020 28709120

[B38] Lyskov S. Gray J. J. (2008). The RosettaDock Server for Local Protein-Protein Docking. Nucleic Acids Res. 36, W233–W238. 10.1093/nar/gkn216 18442991 PMC2447798

[B39] Marianayagam N. J. Sunde M. Matthews J. M. (2004). The Power of Two: Protein Dimerization in Biology. Trends Biochem. Sci. 29, 618–625. 10.1016/j.tibs.2004.09.006 15501681

[B40] Mei G. Di Venere A. Rosato N. Finazzi-Agrò A. (2005). The Importance of Being Dimeric. FEBS J. 272, 16–27. 10.1111/j.1432-1033.2004.04407.x 15634328

[B41] Miyake-Stoner S. J. Refakis C. A. Hammill J. T. Lusic H. Hazen J. L. Deiters A. (2010). Generating Permissive Site-specific Unnatural Aminoacyl-tRNA Synthetases. Biochemistry 49, 1667–1677. 10.1021/bi901947r 20082521

[B42] Mou Y. Huang P.-S. Hsu F.-C. Huang S.-J. Mayo S. L. (2015). Computational Design and Experimental Verification of a Symmetric Protein Homodimer. Proc. Natl. Acad. Sci. USA 112, 10714–10719. 10.1073/pnas.1505072112 26269568 PMC4553821

[B43] Nooren I. M. A. Thornton J. M. (2003). NEW EMBO MEMBER'S REVIEW: Diversity of Protein-Protein Interactions. EMBO J. 22, 3486–3492. 10.1093/emboj/cdg359 12853464 PMC165629

[B44] Norn C. H. André I. (2016). Computational Design of Protein Self-Assembly. Curr. Opin. Struct. Biol. 39, 39–45. 10.1016/j.sbi.2016.04.002 27127996

[B45] Onoda A. Kakikura Y. Uematsu T. Kuwabata S. Hayashi T. (2012). Photocurrent Generation from Hierarchical Zinc-Substituted Hemoprotein Assemblies Immobilized on a Gold Electrode. Angew. Chem. Int. Ed. 51, 2628–2631. 10.1002/anie.201105186 22509501

[B46] Oohora K. Fujimaki N. Kajihara R. Watanabe H. Uchihashi T. Hayashi T. (2018). Supramolecular Hemoprotein Assembly with a Periodic Structure Showing Heme-Heme Exciton Coupling. J. Am. Chem. Soc. 140, 10145–10148. 10.1021/jacs.8b06690 30067348

[B47] Oohora K. Hayashi T. (2014). Hemoprotein-based Supramolecular Assembling Systems. Curr. Opin. Chem. Biol. 19, 154–161. 10.1016/j.cbpa.2014.02.014 24658057

[B48] Pédelacq J.-D. Cabantous S. Tran T. Terwilliger T. C. Waldo G. S. (2006). Engineering and Characterization of a Superfolder green Fluorescent Protein. Nat. Biotechnol. 24, 79–88. 10.1038/nbt1172 16369541

[B49] Pope J. R. Johnson R. L. Jamieson W. D. Worthy H. L. Kailasam S. Ahmed R. D. (2020). Association of Fluorescent Protein Pairs and its Significant Impact on Fluorescence and Energy Transfer. Adv. Sci. 8, 2003167. 10.1002/advs.202003167 PMC7788595 33437587

[B50] Radford R. J. Lawrenz M. Nguyen P. C. Mccammon J. A. Tezcan F. A. (2011). Porous Protein Frameworks with Unsaturated Metal Centers in Sterically Encumbered Coordination Sites. Chem. Commun. 47, 313–315. 10.1039/c0cc02168g 20740227

[B51] Reddington S. C. Baldwin A. J. Thompson R. Brancale A. Tippmann E. M. Jones D. D. (2015). Directed Evolution of GFP with Non-natural Amino Acids Identifies Residues for Augmenting and Photoswitching Fluorescence. Chem. Sci. 6, 1159–1166. 10.1039/c4sc02827a 29560203 PMC5811120

[B52] Reddington S. C. Rizkallah P. J. Watson P. D. Pearson R. Tippmann E. M. Jones D. D. (2013). Different Photochemical Events of a Genetically Encoded Phenyl Azide Define and Modulate GFP Fluorescence. Angew. Chem. Int. Ed. 52, 5974–5977. 10.1002/anie.201301490 23620472

[B53] Reddington S. C. Tippmann E. M. Dafydd Jones D. (2012). Residue Choice Defines Efficiency and Influence of Bioorthogonal Protein Modification via Genetically Encoded Strain Promoted Click Chemistry. Chem. Commun. 48, 8419–8421. 10.1039/c2cc31887c 22801454

[B54] Remington S. J. (2011). Green Fluorescent Protein: a Perspective. Protein Sci. 20, 1509–1519. 10.1002/pro.684 21714025 PMC3190146

[B55] Robinson C. R. Liu Y. Thomson J. A. Sturtevant J. M. Sligar S. G. (1997). Energetics of Heme Binding to Native and Denatured States of Cytochrome B562. Biochemistry 36, 16141–16146. 10.1021/bi971470h 9405047

[B56] Schindelin J. Arganda-Carreras I. Frise E. Kaynig V. Longair M. Pietzsch T. (2012). Fiji: an Open-Source Platform for Biological-Image Analysis. Nat. Methods 9, 676–682. 10.1038/nmeth.2019 22743772 PMC3855844

[B57] Schoffelen S. Beekwilder J. Debets M. F. Bosch D. Hest J. C. M. v. (2013). Construction of a Multifunctional Enzyme Complex via the Strain-Promoted Azide-Alkyne Cycloaddition. Bioconjug. Chem. 24, 987–996. 10.1021/bc400021j 23713411

[B58] Scott D. J. Gunn N. J. Yong K. J. Wimmer V. C. Veldhuis N. A. Challis L. M. (2018). A Novel Ultra-stable, Monomeric Green Fluorescent Protein for Direct Volumetric Imaging of Whole Organs Using CLARITY. Sci. Rep. 8, 667. 10.1038/s41598-017-18045-y 29330459 PMC5766548

[B59] Seifert M. H. J. Georgescu J. Ksiazek D. Smialowski P. Rehm T. Steipe B. (2003). Backbone Dynamics of Green Fluorescent Protein and the Effect of Histidine 148 Substitution†. Biochemistry 42, 2500–2512. 10.1021/bi026481b 12614144

[B60] Song W. J. Tezcan F. A. (2014). A Designed Supramolecular Protein Assembly with *In Vivo* Enzymatic Activity. Science 346, 1525–1528. 10.1126/science.1259680 25525249

[B61] Takeda S. Kamiya N. Arai R. Nagamune T. (2001). Design of an Artificial Light-Harvesting Unit by Protein Engineering: Cytochrome B562-Green Fluorescent Protein Chimera. Biochem. Biophys. Res. Commun. 289, 299–304. 10.1006/bbrc.2001.5966 11708816

[B62] Takeda S. Kamiya N. Nagamune T. (2003). A Novel Protein-Based Heme Sensor Consisting of green Fluorescent Protein and Apocytochrome B562. Anal. Biochem. 317, 116–119. 10.1016/s0003-2697(03)00096-4 12729608

[B63] Thomas S. K. Jamieson W. D. Gwyther R. E. A. Bowen B. J. Beachey A. Worthy H. L. (2020). Site-Specific Protein Photochemical Covalent Attachment to Carbon Nanotube Side Walls and its Electronic Impact on Single Molecule Function. Bioconjug. Chem. 31, 584–594. 10.1021/acs.bioconjchem.9b00719 31743647

[B64] Thomson A. R. Wood C. W. Burton A. J. Bartlett G. J. Sessions R. B. Brady R. L. (2014). Computational Design of Water-Soluble α-helical Barrels. Science 346, 485–488. 10.1126/science.1257452 25342807

[B65] Torres-Kolbus J. Chou C. Liu J. Deiters A. (2014). Synthesis of Non-linear Protein Dimers through a Genetically Encoded Thiol-Ene Reaction. PLoS One 9, e105467. 10.1371/journal.pone.0105467 25181502 PMC4152134

[B66] White C. J. Bode J. W. (2018). PEGylation and Dimerization of Expressed Proteins under Near Equimolar Conditions with Potassium 2-Pyridyl Acyltrifluoroborates. ACS Cent. Sci. 4, 197–206. 10.1021/acscentsci.7b00432 29532019 PMC5833003

[B67] Willis K. J. Szabo A. G. Zuker M. Ridgeway J. M. Alpert B. (1990). Fluorescence Decay Kinetics of the Tryptophyl Residues of Myoglobin: Effect of Heme Ligation and Evidence for Discrete Lifetime Components. Biochemistry 29, 5270–5275. 10.1021/bi00474a008 2383545

[B68] Wittung-Stafshede P. Lee J. C. Winkler J. R. Gray H. B. (1999). Cytochrome B562 Folding Triggered by Electron Transfer: Approaching the Speed Limit for Formation of a four-helix-bundle Protein. Proc. Natl. Acad. Sci. 96, 6587–6590. 10.1073/pnas.96.12.6587 10359755 PMC21958

[B69] Worthy H. L. Auhim H. S. Jamieson W. D. Pope J. R. Wall A. Batchelor R. (2019). Positive Functional Synergy of Structurally Integrated Artificial Protein Dimers Assembled by Click Chemistry. Commun. Chem. 2, 83. 10.1038/s42004-019-0185-5

[B70] Yeates T. O. Liu Y. Laniado J. (2016). The Design of Symmetric Protein Nanomaterials Comes of Age in Theory and Practice. Curr. Opin. Struct. Biol. 39, 134–143. 10.1016/j.sbi.2016.07.003 27476148

[B71] Zacharias D. A. Violin J. D. Newton A. C. Tsien R. Y. (2002). Partitioning of Lipid-Modified Monomeric GFPs into Membrane Microdomains of Live Cells. Science 296, 913–916. 10.1126/science.1068539 11988576

[B72] Zaki A. J. Hartley A. M. Reddington S. C. Thomas S. K. Watson P. Hayes A. (2018). Defined Covalent Assembly of Protein Molecules on Graphene Using a Genetically Encoded Photochemical Reaction Handle. RSC Adv. 8, 5768–5775. 10.1039/c7ra11166e PMC9078156 35539607

