# Supplementary material for: Designed Artificial Protein Heterodimers With Coupled Functions Constructed Using Bio-Orthogonal Chemistry
Source: Front Chem. 2021 Aug 4;9:733550. doi: 10.3389/fchem.2021.733550 (PMC8371201; doi:10.3389/fchem.2021.733550)
Supplement: Supplementary file 1 [file DataSheet1.DOCX]

Supplementary Material

# Supplementary Figures and Tables

**Supplementary Table 1.** Ranked Placement heterodimer models by RosettaDock.

| **Ranked Placement** | **Model ID** | **Model Total Energy**  **(kJ/mole)** | **Interface Energy**  **(kJ/mole)** |
| --- | --- | --- | --- |
| 1 | RD1 | -358.2 | -9.632 |
| 2 | RD2 | -354.0 | -9.091 |
| 3 | RD8 | -353.4 | -7.078 |
| 4 | RD9 | -352.9 | -10.024 |
| 5 | RD7 | -350.5 | -8.83 |

**Supplementary Table 2.** Molar absorbance coefficients for monomers and heterodimers.

| **Peak** | **Molar absorbance coefficient (M^-1^ cm^-1^)** | | | | |
| --- | --- | --- | --- | --- | --- |
|  | **Monomer** | | | **Dimer** | |
|  | **sfGFP^148SCO^** | **cyt *b*_562_^71azF^** | **sfGFP^204SCO^** | **GFP*b*^148-71^** | **GFP*b*^204-71^** |
| Haem soret peak | - | 105,200 | - | 120,300 | 99,300 |
| sfGFP chromophore | 31,000/17,300  (395 nm/492nm) | - | 39,800 | 50,900 | 73,100 |

**Supplementary Table 3.** Molar absorbance coefficients for GFP*b*^204-71^ under different redox conditions.

|  | **Molar absorbance coefficients (M^-1^cm^-1^)**  **(λ in brackets)** | | | |
| --- | --- | --- | --- | --- |
|  | **Soret Peak** | **sfGFP chromophore** | **α Peak** | **β Peak** |
| **Oxidised** | 91,000  (418 nm) | 66,000  (485 nm) | 9,000  (531 nm) | 6,000  (561.5 nm) |
| **Reduced** | 14,000  (426.5 nm) | 65,000  (485 nm) | 13,000  (531 nm) | 11,000  (561.5 nm) |

**Supplementary Figure 1**. SPAAC crosslink at a cyt *b*_562_ residue outside the predicted compatible interface. (a) chemical structure of the two non-canonical amino acids used, *p*-azido-L-phenyalalanine (AzF) and s-cyclooctyne-L-lysine (SCO-K) (a) Structure of cyt *b*_562_ with residue 50 modelled as azF. (b) SPAAC reaction between sfGFP^204SCO^ and cyt *b*_562_^50azF^ as analysed by SDS PAGE.

**Supplementary Figure 2.** The top 10 interface predictions as ranked by ClusPro. Shown are the top 10 models of sfGFP (PDB 2b3p; Green) and cyt *b*_562_ (PDB 1qpu; red). The models are ranked according to their cluster size, with the cluster sizes shown in the accompanying table.

**Supplementary Figure 3.** Spectral characterisation of cyt *b*_562_^71azF^. Top is the absorbance spectra of the wild-type (dashed line) and 71azF variant (sold line) with the calculated molar absorbance coefficients at 418 nm shown in the table below.

**Supplementary Figure 4.** SDS-PAGE analysis of SPAAC between sfGFP and cyt *b*_562_. The relative positions of the monomeric sfGFP (26 kDa), cyt *b*_562_ (17 kDa) and the dimer (39 kDa) are indicated on the diagram. Shown is SDS-PAGE of the SPAAC between (a) sfGFP^148SCO^ or (b) sfGFP^204SCO^ and cyt *b*_562_^71azF^.

**Supplementary Figure 5.** The effect of (a) free haem and (b) free cyt *b*_562_ on the fluorescence emission of sfGFP. (a) Emission of 1 μM sfGFP on excitation at 485 nm in the absence (green solid line) and presence (red dashed line) of 1 μM haem. (b) Emission of 1 μM sfGFP on excitation at 485 nm in the absence (green solid line) and presence (red dashed line) of 1 μM cyt *b*_562_.

**Supplementary Figure 6.** SDS PAGE of fractions separated by size exclusion chromatography of the reaction between cyt *b*_562_^71AzF^ and sfGFP^204SCO^_._

**Supplementary Figure 7.** Emission profile of sfGFP (0.5μM) in the absence (black line) and presence of 1 mM DTT (2000 fold molar excess). Emission was measured on excitation at 485 nm and normalised to the sfGFP emission in the absence of reducing agent.
